# Commentary on Pancreatic Carcinoma: The Role of Radiofrequency Ablation in Advanced Disease

**DOI:** 10.3390/cancers2042055

**Published:** 2010-12-08

**Authors:** John Spiliotis

**Affiliations:** Department of Surgery, “METAXA” Cancer Hospital, Piraeus, 51 Botassi Street, Piraeus, Greece; E-Mail: jspil@in.gr; Tel.: +30-6042-404014

**Keywords:** pancreatic cancer, ablation

## Abstract

Some comments about the role of ablation techniques in the management of advanced pancreatic cancer as palliative procedure.

Pancreatic cancer is the fifth cause of cancer death in the United States and one of the leading causes of cancer death in Western countries, becoming so a major worldwide public health problem.

The only beneficial procedure concerning the long term survival of patients with pancreatic cancer remains the R0 pancreatic resection [1]. Unfortunately, at the time of diagnosis the majority of patients have unresectable tumors due to locally advanced disease, especially in the liver. Treatment options for advanced unresectable pancreatic cancer are very limited. Palliation involves either biliary stenting by ERCP (Endoscopic Retrograde CholedochoPancreatography) or surgical bypass.

Chemotherapy or chemoradiation confers symptomatic improvement in these patients.

RFA (RadioFrequency Ablation) is a local ablative method that has been used for the last five years in different centers with controversial and some attractive results [2,3,4].

Radiofrequency energy has been used in the last decades for the destruction of solid tumors. Unresectable liver tumors, mainly metastases from colorectal cancer, are the primary indication for the method. Promising results have also been reported for many other tumors such as early stage breast cancer, osteoid osteoma, osseous metastases, solid renal tumors, pulmonary malignancies, brain and prostate cancer.

The review by Pezzilli *et al.* on this topic, appearing in *Cancers* [6], offers an attractive article. The authors revised the literature; however, I disagree with their final conclusion and the title as “RFA is not a correct approach for advanced unresectable ductal pancreatic carcinoma”. The main problem concerning the use of RFA in advanced pancreatic cancer disease is the experience of the center which performs the method.

It is well known that the RFA method still demands more investigation before we can respond to general questions about the optimal and most beneficial approach. First, we need prospective studies with homogenous populations at the same stage of tumor development, with similar location of the tumor and also the same surgical approach but without radiological approach.

However, all studies published until now have demonstrated that RFA is a feasible technique in patients with advanced pancreatic cancer, without intra-operative mortality.

In our department we performed this method with excellent results. In a preliminary report three years ago, we demonstrated a benefit in survival rate when compared with a group of patients in which we performed only palliative surgery [4].

When this benefit was calculated for patients of the same stage of cancer development, RFA provided a survival benefit, especially in stage III pancreatic cancer patients.

Most recently, our results were confirmed also by Girelly *et al.* [5], with 50 patients demonstrating acceptable morbidity and mortality; these excellent results suggest the method as an alternative in well selected patients with advanced pancreatic disease.

Another important factor concerning the use of RFA in advanced pancreatic cancer is that all studies must be validated not only for the feasibility and safety but also for the long term survival in combination with other palliative treatments such as adjuvant chemotherapy or chemo radiation.

The article by Pezzilli *et al.* [6] offers an attractive criticism. It indicates that the pooled data of all the included studies are critically influenced by the nature of their constituent reports and methodological problems, such as the lack of randomized trials, end point targets, *etc*. [6]. This is in fact the major problem of all the preliminary results included.

However, on the other hand, the RFA method is in the investigational phase and the main reason for the biotechnology to examine the demands of new instrumentation, *i.e.* specialized electrodes.

As far the thermal kinetic parameters (temperature and time) are concerned, the most important factors, in order to avoid complications, are to apply a temperature of 80–90 °C for a period of 4–6 minutes in one or two session as described in our method [4] and demonstrated by Date in 2005 in a porcine model [7].

At this temperature, ablation of the pancreatic tissue was observed without injury to the adjacent viscera.

In conclusion the RFA treatment is not a standardized approach but it is well tolerated when performed by experienced surgical teams and may provide beneficial survival and palliative benefits (decreased pain) benefits for patients with advanced pancreatic tumors of the body and tail of the pancreas. It is important to re-evaluate the use of RFA treatment in the head of the pancreas with new instruments. Currently this remains a controversial procedure in pancreatic surgery, although under debate.
